# A case of multiple Vertebrobasilar artery fenestration misdiagnosed as vertebral artery dissection

**DOI:** 10.1186/s12883-020-01642-2

**Published:** 2020-02-20

**Authors:** Feng Wang, Xiaokai Wang, Xiaohua Li, Huifeng Zheng, Zhiyong Zhang

**Affiliations:** 1 Department of Neurology, Seventh People’s Hospital of Shanghai, University of Traditional Chinese Medicine, Shanghai, China; 2 Department of Neurology, She Country Hospital, Hebei, China; 3 Department of Neurology, China-Japan Friendship Hospital, Beijing, China

**Keywords:** Vertebrobasilar system, Fenestration, Vertebral artery dissection (VAD), Anomaly, Arterial variation

## Abstract

**Background:**

Fenestration of vertebra-basilar artery is an uncommon congenital vascular anomaly, which is identified by incidental findings on imaging in patients presented without related symptoms or in patients with intracerebral hemorrhage secondary to concomitant artery aneurysm or arteriovenous malformations. Yet, cases of fenestration being misdiagnosed as cerebral artery dissection have never been reported.

**Case presentation:**

We present a patient of 66-year-old female with acute onset of dizziness after chiropractic manipulation of the neck. Neck computed tomography angiography (CTA) showed ‘double lumen’ sign and ‘intimal flap’ of the V1 segment of the vertebral artery, which led to the initial diagnosis of vertebral artery dissection (VAD). However, vertebral artery fenestration at V1 segment was eventually identified by multi-directional digital subtraction angiography (DSA). Interestingly, concomitant vertebral fenestration at V3 segment, basilar fenestration and basilar artery tip aneurysm was also revealed by DSA.

**Conclusion:**

The triple fenestration at vertebrobasilar artery with basilar tip artery aneurysm is extremely rare, and the fenestration at the V1 segment of vertebral artery was easily misdiagnosed as VAD due to the similar imaging morphology.

## Background

Arterial fenestration represents a vessel with a single origin divided into two separate channels and distally converged, which mainly due to incomplete fusion of primitive embryological vessels [1]. Fenestration is a rare developmental vascular anomaly that can occur in multiple cerebral arteries, with vertebrobasilar fenestration being commonly reported [1, 2]. Proximal basilar trunk and extracranial segments of the vertebral artery at the upper cervical level are more commonly seen in basilar and vertebral artery fenestration (VAF), respectively [3, 4]. Fenestration of the V1 segment of the vertebral artery, however, is an extremely rare presentation [5]. Clinical significance of arterial fenestration remains indeterminate. It has solely been recognized as an anatomical variation that ought to be cautious to avoid iatrogenic injuries during endovascular diagnostic and therapeutic interventions, and associated with multiple concomitant vascular anomalies. Cases of VAF being misdiagnosed as dissection has not been reported previously.

Vertebral artery dissection (VAD) is an important etiology of posterior circulation ischemic stroke, Vascular imaging is used to confirm an initial diagnosis and to guide serial treatment decisions [6, 7]. Dissection has a range of presentations on vascular imaging studies, with ‘double lumen’ and ‘intimal flap’ being its characteristic feature [8–10], However, these characteristic radiological features are not exclusive to arterial dissection. An example of this is carotid web, which also shows ‘double lumen’ and ‘intimal flap’ appearance on computed tomography angiography (CTA) axial view [11]. In this case, we present a patient with dizziness after chiropractic manipulation of the neck, who was misdiagnosed with dissection of the extracranial segment of the vertebral artery by CTA. Further imaging investigation by digital subtraction angiography (DSA) prompted a final diagnosis of vertebral artery fenestration of the V1 segment with other multiple concomitant vascular abnormalities.

### Case presentation

**Fig. 1:**
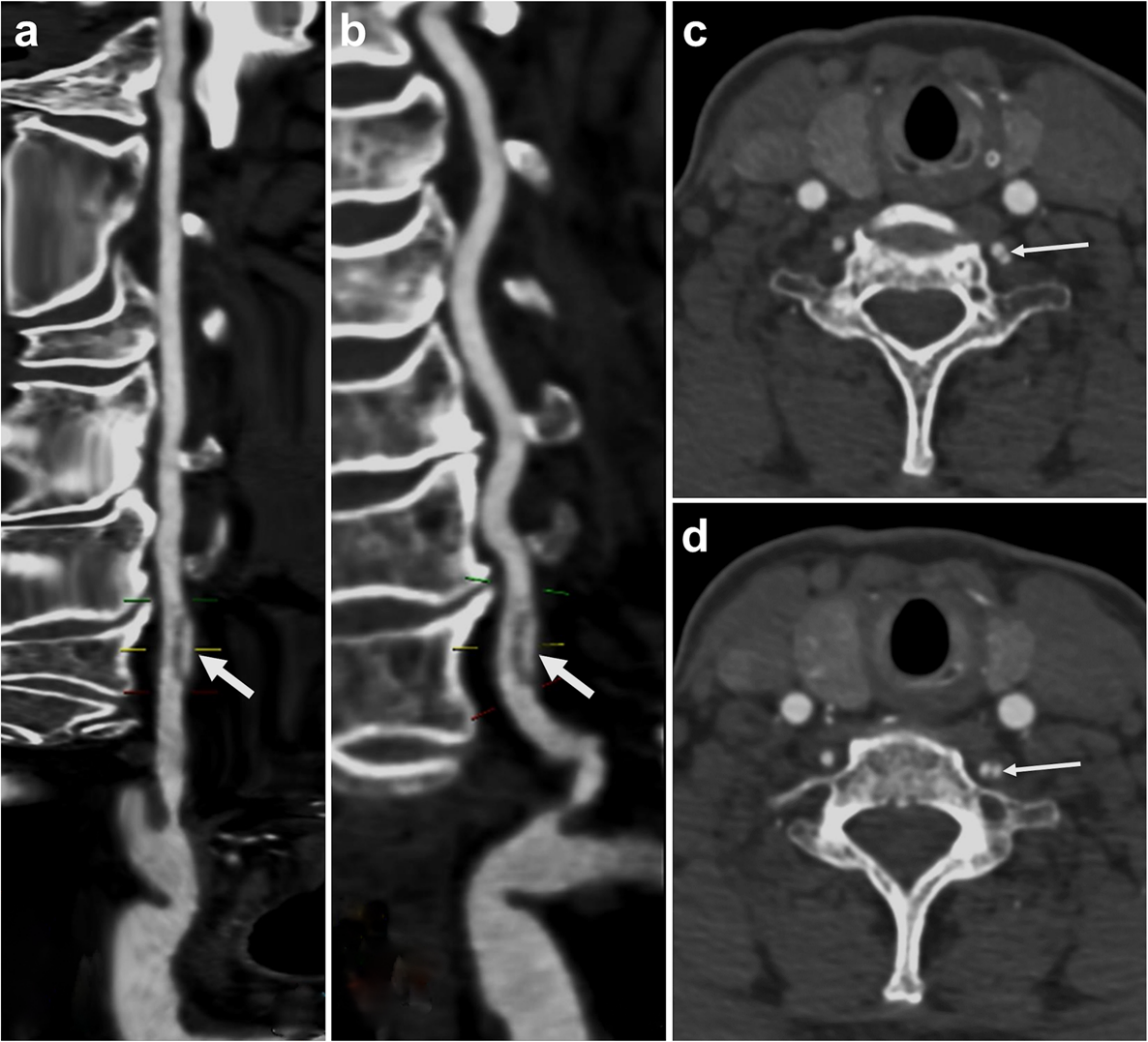
The ‘double lumen’ sign and ‘intimal flap’ in CTA. CTA reconstruction images showing the ‘double lumen’ appearance at the V1 segment of the left vertebral artery (thick white arrows) (**a-b**). CTA axial images showing ‘double lumen’ sign and ‘intimal flap’ (thin white arrows) (**c-d**). CTA = Computed tomography angiography

**Fig. 2:**
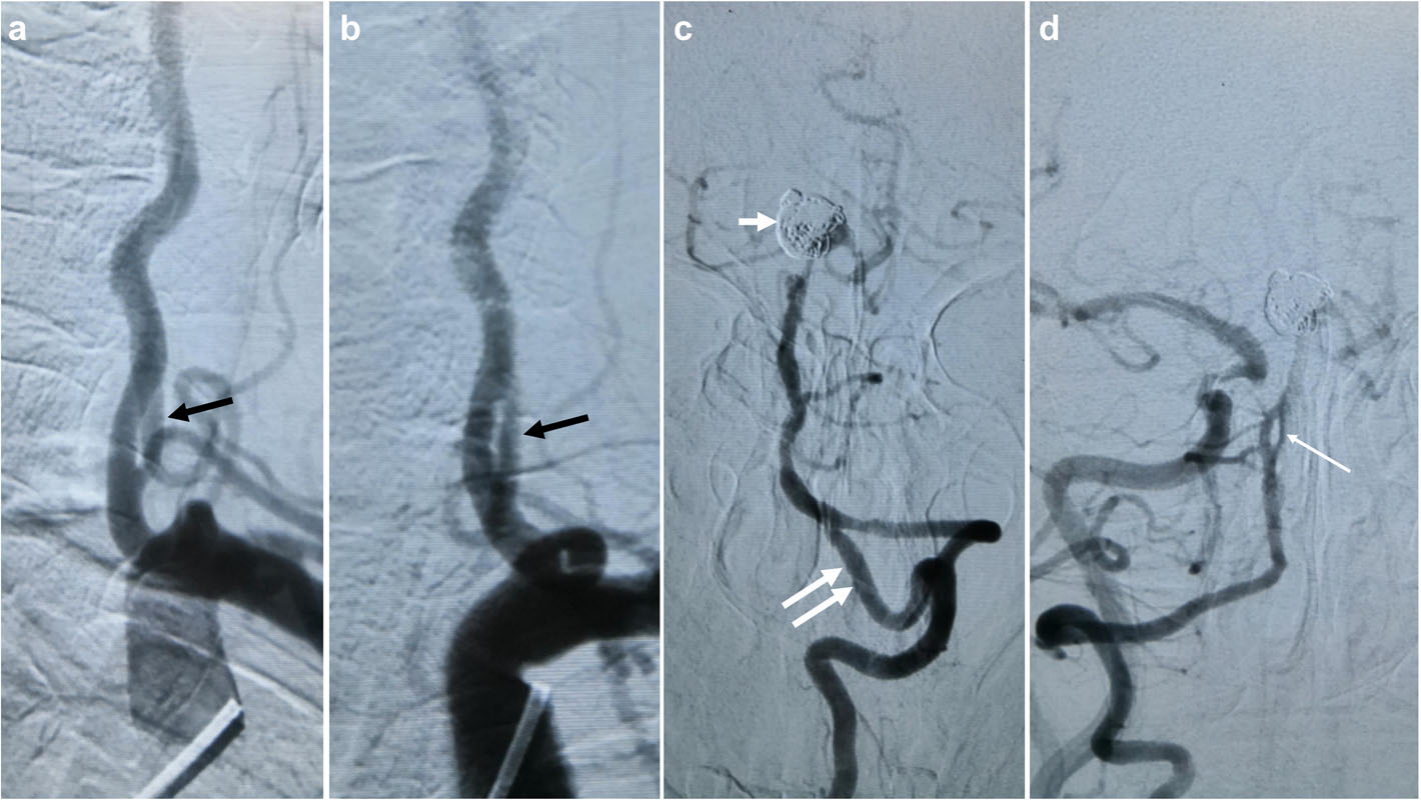
Multiple vascular abnormalities from the vertebrobasilar system in DSA. Extracranial DSA showing the vertebral artery fenestration at the V1 segment in the multiply direction (black arrows) (**a-b**). Intracranial DSA showing an ipsilateral large vertebral artery fenestration at the level of C1 and C2 (double white arrows), post-coil-embolization changes of giant aneurysm at the tip of basilar artery (small white arrow), and a small fenestration at the proximal basilar artery (long white arrow) (**c-d**). DSA = Digital subtraction angiography

A 66-year-old female presented with 2-h history of intermittent dizziness after chiropractic manipulation of the neck, with no spinning sensation, nausea and vomiting. Systemic review of the neurological system after admission to the local hospital suggested no definitive positive signs, except high blood pressure (170/90 mmHg). Past medical history includes a 30-year history of hypertension with poor compliance to antihypertensives, and subarachnoid hemorrhage secondary to basilar artery aneurysm which was treated with coil embolization in another hospital 5 years ago. Because the patient lost the image data of artery aneurysm embolization, no other information about the cerebrovascular condition could be gotten. On the day after admission, in order to identify the cause of dizziness, Cranial computed tomography (CT) and magnetic resonance imaging (MRI) were used to exclude hemorrhagic stroke and new infarction, and no abnormalities were found in the vestibular function test. The next day, the neck CTA showed the ‘double lumen’ and ‘intimal flap’ appearance at V1 segment of the left vertebral artery, which was considered to be VAD (Fig. 1). This patient was transferred to our hospital on the third day for DSA, and the multi-directional DSA excluded the arterial dissection of the V1 segment, and diagnosed as a rare VAF (Fig. 2a-b). In addition, this patient was accompanied by an ipsilateral long VAF at the level of C1 and C2, post-coil-embolization changes of giant aneurysm at the tip of basilar artery and a small fenestration at the proximal basilar artery (Fig. 2c-d). The patient was eventually diagnosed as hypertension-related dizziness and was kept on conservative management with antihypertensives and antivertiginous medications. Her symptoms were diminished completely on the next day after admission and thrombolytic agents were not given. The patient was discharged 2 weeks later with no recurrence during her hospitalization.

## Discussion and conclusions

Vertebrobasilar artery fenestration is an uncommon vascular anomaly in the posterior circulation observed at autopsies and on angiography studies, which is a result of incomplete fusion of primitive embryological arteries [12]. Basilar artery fenestration is more commonly seen in its proximal part [3]. In contrast, VAF can be seen in the intracranial or extracranial segment of the vertebral artery. It is most common in the extracranial segment of the upper cervical level [4], but the V1 segmental fenestration of the vertebral artery is extremely rare, and Gard et al. [5] reported the first such patient in the world in 2013. A few studies have reported that double fenestration can occur in vertebral artery or basilar artery [3, 13], and Sogawa et al. [3] suggested the incidence of basilar artery double fenestration was just 0.018%. Additionally, the incidence of vertebral artery combined with basilar artery fenestration is even rarer [3, 12], with only one case being identified in a cohort of 215 patients with basilar artery fenestration in a magnetic resonance angiography (MRA) study of a total of 16,416 patients [3]. Our patient has an extremely rare presentation of triple concomitant fenestration, which hasn’t been reported previously.

Vertebrobasilar artery fenestration is commonly associated with other vascular abnormalities, especially cerebral aneurysm and arteriovenous malformation [14–16]. The combined basal aneurysm is most often reported at the junction of the vertebral-basal artery or at the site of basilar artery fenestration [16–18]. The medial defects of the vessel wall at either end of the fenestrated segment and hemodynamic stress are thought to be the causes of aneurysm formation at these sites [16–18]. In this case, as our patient has basilar tip artery aneurysm, this suggests its formation is more likely to be of congenital origin rather than hemodynamic disturbance. Despite the relevant risk of developing other vascular or tissue abnormalities and the anatomical variation that needs to be considered by operator prior to endovascular intervention or open surgery, the clinical value of arterial fenestration remains indeterminate and requires further investigation. The clinical significance in this case is the misdiagnosis of V1 segment fenestration as VAD.

VAD refers to a disease with a range of etiologies which results in blood flowing into arterial wall from the damaged intima, causing intramural hematoma formation, arterial stenosis, occlusion or rupture [8]. CTA is a sensitive and accurate imaging technique for diagnosing arterial dissection [7, 9]. A study comparing the diagnostic accuracy between multi-slice spiral CTA (MSCTA) and DSA for extracranial VAD indicated MSCTA has a sensitivity of 100%, a specificity of 98% and a positive predictive value of 98.5% [19]. Imaging characteristics of arterial dissection include: 1). pearl and string sign: eccentric arterial stenosis with external diameter expansion; 2). double lumen and intimal flap; 3). string sign: tapered stenosis or occlusion; 4). dissecting aneurysm [9, 10]. Among them, double lumen and intimal flap are the direct signs, which can be used as the main diagnostic basis for arterial dissection [9, 10]. Therefore, given a background of suspected trauma was provided in patient’s history and depiction of double lumen appearance on CTA, the diagnosis of ‘VAD’ was initially easily made. The appearance of double lumen in dissection is formed as a consequence of intimal flap separating the true and false lumen. True lumen is identified as the incompletely occluded vessel lumen with adequate filling of contrast agent and fast hemodynamic flow, resulting in higher density; in contrast, false lumen has the slow hemodynamic flow and therefore slow contrast filling, depicting a lower density on CTA. Yet, density difference between true and false lumen is sometimes difficult to differentiate, and intimal flap in cases like VAD is a relatively difficult feature to be depicted on imaging studies [20]. Teasdale E et al. suggested 45 patients with VAD didn’t have double lumen appearance on CTA and intimal flaps in these cases were too small to be seen [20].

Arterial fenestration is essentially the luminal division of a single-origin vessel into two separate channels anywhere along its course with a distal convergence. These two channels have relatively complete arterial wall structures, each consisting of endothelial and muscular layers with or without a shared adventitia [4, 21]. In contrast to basilar artery fenestration, the two separate limbs of VAF are usually wide apart and form a convex-lens-like shape [22], therefore, making it a straightforward diagnosis even on CTA/ MRA. In this case, our patient has a short fenestration at the segment of V1, and the two limbs are in proximity, forming a slit-like shape and resulting in no contrast filling on CTA. Hence it was easily misdiagnosed as ‘intimal flap of a dissection’. Additionally, if the two channels are situated side by side, they can be confused with double lumen appearance on CTA. In this case, multidirectional DSA depicted the conspicuous view of two channels of the vertebral artery separating and converging, with consistency of contrast staining around the suspected lesion and no contrast stagnation. And, the density values or CT values of the two limbs of fenestration were the same according our retrospective measurement. The final diagnosis of VAF was made, and due to our timely diagnosis, the immeasurable iatrogenic injuries of misuse of anti-thrombotic agents or endovascular intervention for “dissection” were avoided.

In conclusion, we reported a patient with extremely rare abnormality of triple fenestration at vertebrobasilar artery with basilar tip artery aneurysm. The fenestration at the V1 segment of vertebral artery was initially misdiagnosed as VAD due to the short segment of fenestration and the proximity of two channels. The significance of this case is that it further expands our knowledge of rare anatomical anomaly of vertebrobasilar artery, the relevant professional doctors can make the correct diagnosis and treatment to avoid the iatrogenic injury when encountering the such lesions according to this case. Moreover, this case increases the differential diagnostic spectrum of extracranial VAD.

## Data Availability

Not applicable.

## Abbreviations

CT: Computed tomography
CTA: Computed tomography angiography
DSA: Digital subtraction angiography
MRA: Magnetic resonance angiography
MRI: Magnetic resonance imaging
MSCTA: Multi-slice spiral CTA
VAD: Vertebral artery dissection
VAF: Vertebral artery fenestration
